# Optimizing Covalent Immobilization of Glucose Oxidase and Laccase on PV15 Fluoropolymer-Based Bioelectrodes

**DOI:** 10.3390/jfb13040270

**Published:** 2022-12-01

**Authors:** Nicolò Montegiove, Eleonora Calzoni, Dario Pelosi, Luca Gammaitoni, Linda Barelli, Carla Emiliani, Alessandro Di Michele, Alessio Cesaretti

**Affiliations:** 1 Department of Chemistry, Biology and Biotechnology, University of Perugia, Via del Giochetto, 06123 Perugia, Italy; 2 Centro di Eccellenza sui Materiali Innovativi Nanostrutturati (CEMIN), University of Perugia, Via Elce di Sotto 8, 06123 Perugia, Italy; 3 Department of Engineering, University of Perugia, Via G. Duranti 93, 06125 Perugia, Italy; 4 Department of Physics and Geology, University of Perugia, Via Pascoli, 06123 Perugia, Italy

**Keywords:** enzymatic biofuel cell, glucose oxidase, laccase, bioanode, biocathode, biomedical devices, enzyme immobilization

## Abstract

Enzymatic biofuel cells (EBCs) represent a promising technology for biosensors, biodevices, and sustainable green energy applications, thanks to enzymes’ high specificity and catalytic efficiency. Nevertheless, drawbacks such as limited output power and short lifetime have to be solved. Nowadays, research is addressed to the use of 3D electrode structures, but the high cost and the industrialization difficulties of such electrodes represent a key issue. The purpose of the paper is thus to describe the use of a low-cost commercial conductive polymer (Sigracell^®^ PV15) as support for the covalent immobilization of glucose oxidase and laccase, for bioanode and biocathode fabrication, respectively. Efficient immobilization protocols were determined for the immobilized enzymes in terms of employed linkers and enzyme concentrations, resulting in significant enzymatic activities for units of area. The analysis focuses specifically on the optimization of the challenging immobilization of laccase and assessing its stability over time. In particular, an optimum activity of 23 mU/cm^2^ was found by immobilizing 0.18 mg/cm^2^ of laccase, allowing better performances, as for voltage output and electrochemical stability, and a direct electron transfer mechanism to be revealed for the fabricated biocathode. This study thus poses the basis for the viable development of low-cost functional EBC devices for biomedical applications.

## 1. Introduction

In recent years, the demand for sustainable means to produce energy has grown enormously. In this framework, among the emerging power supply technologies, biological fuel cells (biofuel cells) may represent an effective power source for several applications, ranging from biomedical devices to waste treatments [1,2,3,4,5,6,7,8,9,10,11,12]. In particular, enzymatic biofuel cells (EBCs) are biofuel cells where the bioelectrochemical system is based on redox enzymes immobilized on electrode surfaces exposed to the reaction environment [13,14,15]. EBCs can be designed to produce energy from various types of simple carbohydrates such as the glucose circulating in the body, but also complex substrates abundantly contained in wastewater [16,17,18,19] which is produced daily in large quantities by various sectors, i.e., industrial, domestic, and agricultural sectors. Specifically, in recent years, there has been an increasing demand for biomedical devices for monitoring and preventing diseases such as heart disease, hypertension, and diabetes [14,20,21]. These systems, after implantation in the body, require battery replacement every ten years through surgery, a convenient and low-risk procedure. If the device requires more power consumption or needs to operate for a longer period, simple primary batteries cannot be used. To satisfy these needs, new systems are required: batteries that can be exploited both as energy sources and for storage purposes, devices that can harvest energy directly from the human body, and systems capable of receiving and operating with the energy transferred from exogenous sources [14]. For these reasons, new power supply technologies are based on high energy density batteries capable of continuously powering devices or recharging their primary batteries, thus reducing the number of surgical procedures required, minimizing the risk of infection, and increasing device reliability. Battery technology, therefore, is a key requirement for developing these biomedical electronic systems, and among these innovative technologies, EBCs may represent a power source for a variety of medical devices due to their ability to generate sufficient power relative to primary power sources. The development of dense energy sources is a major challenge to realizing the next generation of personalized biomedical electronics that are multifunctional, compact, and long-lasting. Further, among their several advantages, EBCs allow operating in moderate electrolytes even under mild conditions, using renewable biocatalysts, and harvesting energy from biological sources or organic pollutants [18].

EBC technology therefore represents a promising tool with the most varied applications, nevertheless, major drawbacks are EBCs’ limited output power, short lifetime, and low efficiency [22]. Such aspects need to be solved before reaching a viable application, with enzyme loading and stability, as well as electron transfer rate, being the most challenging parts of the improvement process [23]. All these issues can be attributable to three main factors: the low loading of the enzyme catalyst, the loss of the enzymes from the electrode, and the slow efficiency of electron transfer between the active center of the enzyme and the electrode. According to the selected enzyme, EBCs can be based on the mediated electron transfer (MET) and the direct electron transfer (DET) mechanisms. The MET mechanism allows higher current densities to be reached relative to DET, because of the use of small electroactive molecules (i.e., mediators) that are able to extract electrons from the active sites of the enzyme and shuttle them from the enzyme to the electrode surface [19,24,25]. However, the thermodynamic redox potential of mediators acts as a system bottleneck leading to the consequent reduction of EBC voltages. Hence, the great interest in EBCs based on the DET mechanism is related to the possibility of reaching high voltage output without the need for mediators to shuttle electrons. The electron transfer mechanism therefore plays a key role in developing EBCs with long-term stability and high-power output, but also the choices concerning the type of enzymes and supports are of the utmost importance. In a typical EBC, enzymes can effectively and specifically catalyze oxidation reactions of fuels (bioanode), and the oxygen reduction reaction (biocathode) under operating conditions [26,27]. Nowadays, the most promising enzymes capable of operating with widely available substrates, also present in human blood (as glucose and oxygen), and transferring electrons through the DET mechanism, are glucose oxidase (GOx) and laccase for the bioanode and biocathode, respectively [8,28,29,30,31,32,33,34,35]. Several studies have been carried out to increase EBCs’ performances by changing the electrode support, for example, by resorting to 3D structures consisting of gold nanoparticles or carbon nanotubes [36,37,38,39]. Nevertheless, although such works showing a strong enhancement in the power density and enzyme lifetime, no commercial production can be carried out because of the high costs and process complexity of these 3D electrodes, which would make their possible use on a large scale very limited. To the best of our knowledge, no research activity has been carried out employing a commercial conductive polymer as support for the EBC electrodes.

Therefore, after a preliminary validation of electrodes realized over polymeric supports in an integrated cell assembly prototype [40], the present paper focuses on the performance enhancement of such bioelectrodes through a suitable tuning of the immobilization parameters and their biochemical and morphological characterization. Specifically, the main aim of this study is to analyze and evaluate the stability and power output of GOx bioanode and laccase biocathode immobilized on commercial support in order to lay the basis for the future development of a long-term stable, high-power, and low-cost EBCs based on the DET mechanism. In particular, this research work concerns the characterization of DET bioelectrodes, immobilizing by covalent ligands GOx and laccase on the commercial conductive fluoropolymer SGL Carbon’s Sigracell^®^ PV15. PV15 is an economic bipolar plate with a 0.6 mm thickness suitable for electrochemical applications, usually employed in flow battery and fuel cell applications. It exhibits good electrical conductivity, low electrical resistivity (<10 Ωmm), and high mechanical resistance (tensile strength of 25 MPa and compressive strength of 170 MPa). It is characterized by a light weight (density of 1.65 g/cm^3^), so that it can be readily available in large dimensions, which makes this fluoropolymer an ambitious low-cost material for several applications [41]. Moreover, a suitable immobilization strategy has been performed and optimized to significantly increase the activity of the immobilized enzymes, preventing their leakage from the considered polymer. Specifically, this study has been mainly focused on the laccase biocathode characterization, which represents the most sensitive and problematic electrode for EBC development, as reported in the literature [42,43,44], although the results of the GOx bioanode characterization are also illustrated for the sake of completeness. Hence, by combining the use of commercial and extremely economical supports with the identification of implemented immobilization procedures, providing the enzyme activity per cm^2^ of the electrode, as extensively described in this paper, could indeed pave the way for EBC application in biomedical devices and their scale-up as alternative energy sources in industrial applications.

## 2. Materials and Methods

### 2.1. Materials

A commercial conductive fluoropolymer Sigracell^®^ PV15, purchased from SGL Carbon (Wiesbaden, Germany), was chosen as the support electrode material for enzyme immobilization. N-(3-Dimethylaminopropyl)-N′-ethylcarbodiimide hydrochloride (EDC), N-Hydroxysuccinimide (NHS), diethylenetriamine, 25% glutaraldehyde stock solution, potassium phosphate, sodium acetate (NaOAc), glucose oxidase from *Aspergillus niger*, peroxidase from horseradish, laccase from *Trametes versicolor*, o-Dianisidine, D-Glucose, and 2,2′-Azino-bis (3-ethylbenzothiazoline-6-sulfonic acid) diammonium salt (ABTS) were purchased from Sigma-Aldrich (Saint Louis, MO, USA).

### 2.2. Methods

#### 2.2.1. Bioanode Preparation: Covalent Immobilization of Glucose Oxidase (GOx)

A functionalization step of the support material PV15 precedes an activation phase in the enzyme immobilization protocol [45,46]. A diethylenetriamine solution diluted in propanol was used to react with PV15, featuring a surface area of 1 cm^2^, for 1 h at room temperature in a FALC ultrasonic bath (FALC Instruments, Treviglio, BG, Italy) in order to functionalize the material with NH_2_ groups. Washings of the PV15 materials with deionized H_2_O were later performed. An activation step was subsequently carried out by using a bifunctional agent capable of binding both to the NH_2_ groups of the material and the NH_2_ groups of the amino acid side chains of the enzyme. PV15 was activated with a 2.5% (*v*/*v*) glutaraldehyde solution in deionized H_2_O for 3 h at room temperature in a FALC ultrasonic bath (FALC Instruments, Treviglio, BG, Italy). After the elimination of the glutaraldehyde solution and extensive washing of the PV15 materials with deionized H_2_O, GOx enzyme was immobilized on the support by placing 500 µL of the enzymatic solutions (1.0 and 0.50 mg/mL in a 50 mM NaOAc solution at pH 5.0) over the PV15 films and incubating overnight at 4 °C on a laboratory shaker (Bibby Stuart Platform Rocker STR6) set at 20 rpm. The determination of GOx concentration linked to PV15 was determined through the glucose oxidase assay [47] by evaluating the difference between the enzymatic activity of the GOx stock solution and that of the solution recovered after the immobilization process. For each GOx concentration tested, the results are the mean of three GOx-PV15 preparations.

#### 2.2.2. Biocathode Preparation: Covalent Immobilization of Laccase

For the immobilization of laccase, the chosen method was based on the combined use of EDC/NHS. The surface of the PV15 material (featuring a surface area of 1 cm^2^) was activated with 500 µL of a mixture containing 0.5 mM EDC and 0.1 mM NHS in deionized H_2_O and eight different concentrations of laccase (from 17.5 mg/mL to 0.020 mg/mL) for at least 24 h at 4 °C on a laboratory shaker (Bibby Stuart Platform Rocker STR6) set at 20 rpm. The determination of laccase concentration linked to PV15 was determined through the laccase assay [48,49,50], by evaluating the difference between the enzymatic activity of the laccase stock solution and that of the solution recovered after the immobilization process. For each laccase concentration tested, the results are the mean of three laccase-PV15 preparations.

#### 2.2.3. SEM Analysis

The surface morphology of the samples was observed by field emission gun electron scanning microscopy (FE-SEM) with an LEO 1525 ZEISS (Carl Zeiss SMT AG, Oberkochen, Germany), metalizing the samples with chromium. The electron high tension (EHT) used was 5 keV and an in-lens detector was used for the secondary electrons.

#### 2.2.4. FT-IR/ATR Measurements

The infrared analyses of the bioelectrodes were carried out by a Fourier transform (FT-IR) Alpha instrument from Bruker Optics GmbH (Ettlingen, Germany) and its attenuated total reflection (ATR) module was equipped with a diamond crystal. The spectra were acquired with 30 scans in the range 4000–400 cm^−1^ with a resolution of 2 cm^−1^.

#### 2.2.5. GOx Enzymatic Assay

**Scheme 1:**
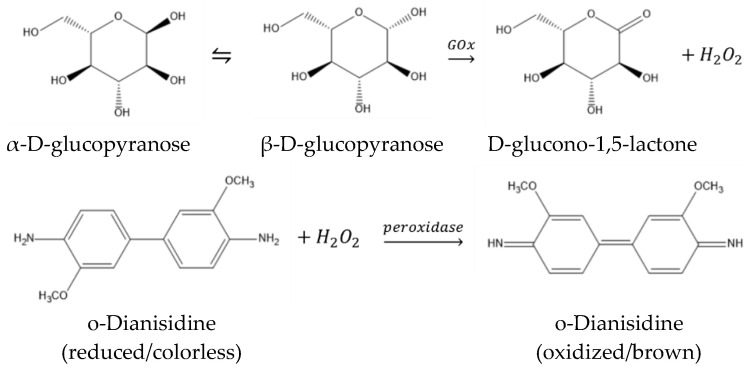
Coupled reactions carried out by glucose oxidase (GOx) and peroxidase in the colorimetric assay.

The enzyme activity of free and immobilized GOx was performed by a colorimetric assay [47], where the increase in absorbance at 460 nm is related to the oxidation of o-Dianisidine. The oxidation of the reagent is attained through a GOx/peroxidase coupled system starting from glucose according to the reactions reported in Scheme 1. The assay was carried out as already described in [40].

#### 2.2.6. Laccase Enzymatic Assay

**Scheme 2:**
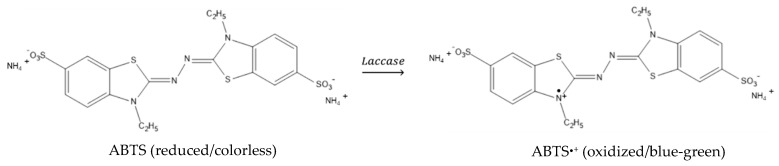
Reaction carried out by laccase in the colorimetric assay.

Laccase activity was determined by the colorimetric assay based on the oxidation of ABTS [48,49,50]. Laccase is capable of oxidizing the colorless dye ABTS to its more stable radical cation, featuring an intense blue-green color following the reaction shown in Scheme 2. The assay was performed according to the method described in detail in [40].

#### 2.2.7. Electrochemical Characterization of Bioanode

A preliminary activity, including open-circuit voltage (OCV) and cyclic voltammetry (CV) tests, was carried out on a bioanode consisting of immobilized GOx on the same commercial support (Sigracell^®^ PV15, SGL Carbon, Wiesbaden, Germany), aiming at evaluating its electroanalytical performance. Specifically, for the GOx bioanode, OCV and CV measurements were performed at different concentrations of glucose (from 0 to 280 mM) in NaOAc pH 5.0 buffer solution. Moreover, CA tests were carried out for the bioanode at varying glucose concentrations as well. Considering the stable value measured for the OCV, as discussed in the Results and Discussion section, no OCV tests over time are reported in this paper for the bioanode. Moreover, it must be emphasized that with the biocathode being the most critical component for developing highly efficient and stable biofuel cells over time, this paper is mainly focused on the laccase biocathode performance evaluation.

#### 2.2.8. Electrochemical Characterization of Biocathode

An experimental procedure was developed and applied to characterize the electrochemical activity of the biocathode over time. To evaluate the electrochemical activity of the biocathode consisting of laccase enzyme immobilized on a commercial flexible graphite support (Sigracell^®^ PV15, SGL Carbon, Wiesbaden, Germany), both cyclic voltammetry (CV) and OCV tests were carried out through the galvanostat/potentiostat BioLogic^®^ SP-240, immersing the working electrode in a NaOAc buffer solution at pH 5.0. The galvanostat/potentiostat SP-240 was connected to a three-electrode electrochemical cell, using a Pt wire counter electrode and a silver/silver chloride reference electrode (Ag/AgCl/KCl_sat_).

CV measurements were carried out by fixing a scan rate equal to 20 mV/s in a range between −1 V and 1 V for all the experiments described, at room temperature. Specifically, CV was performed:

1. In phosphate buffer solution after purging nitrogen for half an hour.
2. In phosphate buffer solution after purging air for at least half an hour.
3. In phosphate buffer solution after purging air and in presence of the catechol mediator.

Subsequently, CA tests were performed to quantify the steady-state current passing through the electrode. Such tests were realized by applying a voltage of 0.1 V vs. Ag/AgCl and registering the current over time with a sample time of 0.1 s. Moreover, to evaluate biocathode stability over time, the OCV was measured over six weeks. In detail, the OCV of the biocathode was registered by leaving the electrode in an air-saturated phosphate buffer solution pH 5.0 for a whole night. Between one test and the other, the electrode was stored at a temperature of 4 °C in a phosphate buffer solution pH 5.0.

## 3. Results and Discussion

GOx and laccase were covalently immobilized on the commercial conductive fluoropolymer SGL Carbon’s Sigracell^®^ PV15 for the bioanode and biocathode fabrication, respectively. When dealing with immobilized enzymes, the parameters that need to be considered are: (a) the immobilization yield, expressed as the percentage of the number of immobilized molecules over the total enzyme that has been placed in contact with the polymer surface; (b) the recovered activity, that is the specific activity (in U/mg) of the immobilized enzyme relative to that of its free form; (c) the enzymatic activity for units of area (in U/cm^2^), that describes the actual functionality of the enzyme-loaded films. These three factors depend on the immobilization conditions employed and were optimized for the development of ad hoc effective immobilization protocols for the two enzymes.

### 3.1. Bioanode Characterization

**Figure 1:**
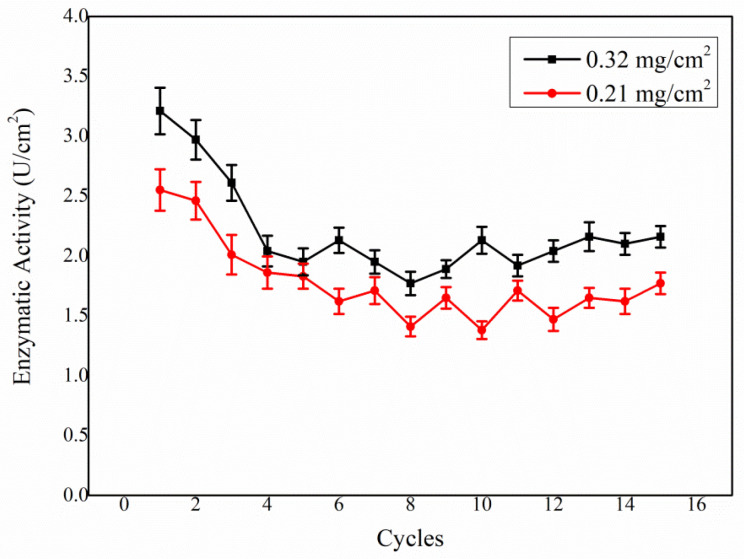
Enzyme activity of GOx immobilized on PV15 films recorded for 15 successive cycles over more than one month. Data are represented as mean ± SEM, n = 3.

The immobilization of GOx was carried out through the procedure described in the Material and Methods section by resorting to an amine and glutaraldehyde for the covalent linkage of the enzyme to the PV15 support. Two different concentrations of GOx were used, namely 1.0 and 0.50 mg/mL, leading to 0.32 (immobilization yield = 64%) and 0.21 (immobilization yield = 84%) mg/cm^2^ of immobilized enzymes, respectively, in line with the typical concentrations reported in the literature for the immobilization of this enzyme [51]. The activity of immobilized GOx was assessed as described in the “GOx Enzymatic Assay” subsection, and values of 3.2 and 2.6 U/cm^2^ were recorded for the two different concentrations, corresponding to specific activities of 10.0 and 12.1 U/mg, respectively. The specific activity of free GOx is 17.3 U/mg, meaning that the enzyme in its immobilized form retains about 60–70% of its activity. The activity of immobilized GOx, which would serve as bioanode, was later tested as a function of time (Figure 1). An initial decrease in activity was detected during the first three/four cycles, following a behavior that had already been observed for other immobilized enzymes [45,46]. Later, after the activity had reduced to about 60% of its initial value, an almost constant activity was measured for many successive cycles, proving the stability of the system.

### 3.2. Biocathode Characterization

**Figure 2:**
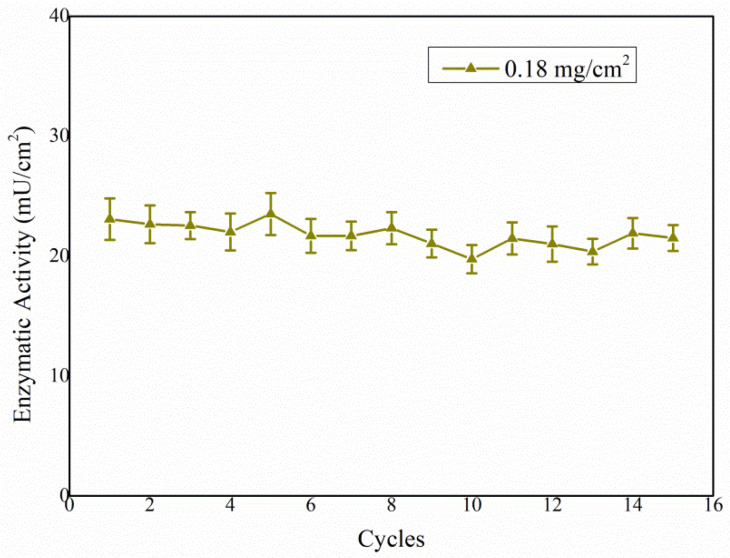
Enzyme activity of laccase immobilized on PV15 films recorded for 15 successive cycles over more than one month. Data are represented as mean ± SEM, n = 3.

Laccase was instead immobilized by resorting to a solution consisting of EDC/NHS. In light of the scarce information on the immobilization of laccase found in the literature, many different concentrations of the enzyme were tested, spanning three orders of magnitude, ranging from 17.5 mg/mL to 0.020 mg/mL. The percentage of immobilization was evaluated by the difference in activity between the starting solution and the one collected after the immobilization had occurred. The immobilization yields are reported in Table 1 for all of the eight films that were prepared. As can be readily seen, the least concentrated solutions led to an immobilization yield greater than 90%, while this value is reduced to 80.5%, 48%, and 42% for 2 mg/mL, 8.75 mg/mL, and 17.5 mg/mL, respectively. This finding is most likely due to the saturation of the polymer surface, which cannot host these large amounts of enzyme molecules. Further to this, the activity of immobilized laccase was also evaluated, and the results are again reported in Table 1. Interestingly, the specific activity of the immobilized enzyme is found to increase as the concentration of immobilized laccase decreases. This concentration-dependent behavior can be explained considering that, under crowding conditions, protein–protein interactions between adjacent immobilized enzymes may hinder the flexibility of the molecule and thus its ability to capture the substrate and release the product of the reaction [52]. The excessive enzyme loading is therefore an issue to be reckoned with. In particular, by reducing the concentration of the immobilized laccase down to 0.010 mg/cm^2^ (film **8**), the recovered specific activity is 0.45 U/mg, which is about 45% of the free enzyme, whereas it decreases by almost three orders of magnitude to 1.1 × 10^−3^ U/mg (being just 0.11% of its free form) in the case of the most concentrated laccase-PV15 film (film **1**, 3.7 mg/cm^2^). Although the highest specific activity was found for the least concentrated films, the greatest activity for units of area, i.e., 23 mU/cm^2^, was measured for the film loaded with a concentration of 0.18 mg/cm^2^ (film **5**). Therefore, the latter is the formulation that was chosen to be later employed for the electrochemical characterization of the biocathode by virtue of its higher enzymatic activity. Film **5** was also tested as a function of time in order to evaluate the stability of the immobilized enzyme. Its functionality was measured for numerous successive cycles and only a minimum loss of activity was recorded after 15 cycles (Figure 2).

### 3.3. Morphological Characterization

**Figure 3:**
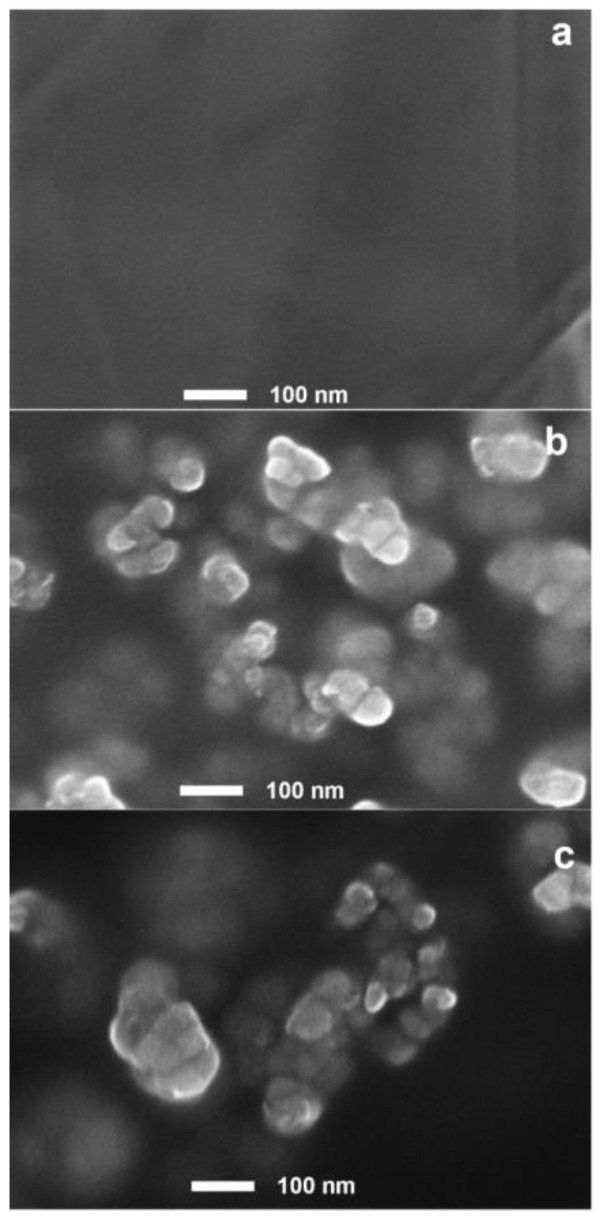
SEM images of PV15 (**a**), PV15 with immobilized GOx to serve as bioanode (**b**), and PV15 with immobilized laccase to serve as biocathode (**c**).

**Figure 4:**
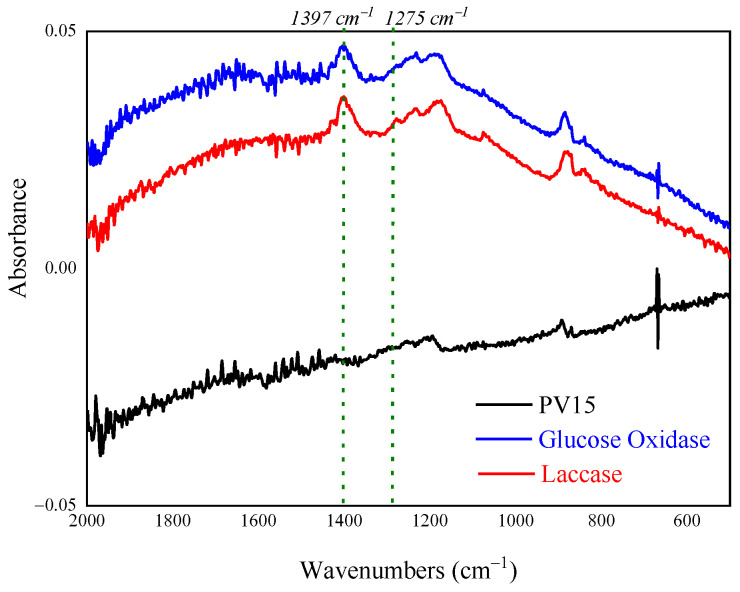
FT-IR/ATR infrared spectra of PV15 (black line), PV15 with immobilized GOx (blue line), and PV15 with immobilized laccase (red line).

SEM and FT-IR/ATR analyses were carried out to verify the binding of the GOx and laccase on PV15 bioelectrodes (Figure 3 and Figure 4).

SEM micrographs of the surface morphology of polymeric support before (Figure 3a) and after enzyme immobilization (Figure 3b,c) confirmed the homogeneous distribution of bound enzymes on the PV15 surfaces. The polymer was observed at such a magnification as to be able to appreciate an acceptable enzymatic distribution (about 1 μm^2^ surface): Figure 3a shows the homogeneous and regular surface of PV15, perfectly adaptable to an enzymatic immobilization process for both protocols and enzymes used. The SEM analysis reported in Figure 3b,c shows instead that the surface morphology of the polymeric support changed upon immobilization. In particular, before the immobilization, the polymeric film had a uniform and crack-free surface (Figure 3a), while after immobilization both bioelectrodes showed irregular and nanostructured surfaces revealing the successful and widespread functionalization of the material (Figure 3b,c). The SEM images of the enzymes immobilized on the PV15 support were acquired on samples after using the immobilized enzymes for several cycles, proving the stability of the bond formed for the given amount of enzyme.

The FT-IR/ATR analysis revealed different infrared spectra for the PV15 surface and the two bioelectrodes, that is to say, the material after the immobilization of the enzymes. Both bioelectrodes exhibited a profile with a characteristic band at 1397 cm^−1^ ascribable to the C−N stretching vibration of a primary amide, whereas the band at 1275 cm^−1^ can be assigned to a mixed vibration involving the OCN and N−H modes of a tertiary amide [53]. These bands confirmed the presence of immobilized enzymes on the PV15 surface.

### 3.4. Electrochemical Analysis

#### 3.4.1. Cyclic Voltammetry

Cyclic voltammetry (CV) is a powerful technique in electroanalytical chemistry that provides direct insights into the kinetics of electrode reactions [54]. According to the procedure described in the Materials and Methods section, the CV experimental tests were conducted aiming at assessing the electrocatalytic activity of the GOx and laccase enzymes immobilized on the chosen commercial support (PV15).

#### 3.4.2. Bioanode Cyclic Voltammetry

**Figure 5:**
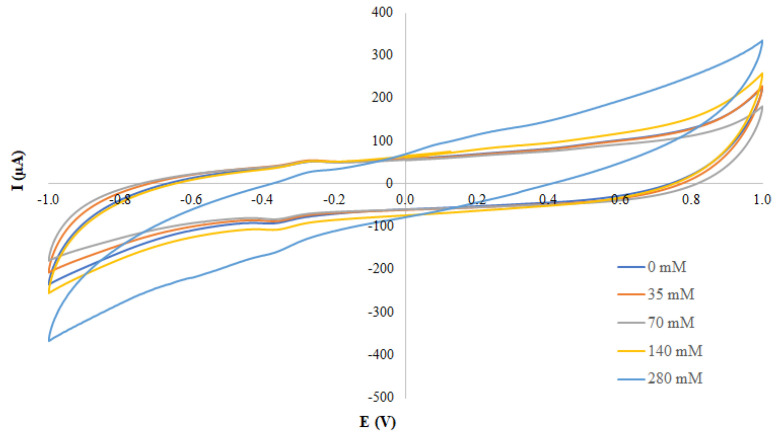
Cyclic voltammetry registered for GOx bioanode in a NaOAc buffer solution (pH 5.0) at different concentrations of glucose: 0 mM, 35 mM, 70 mM, 140 mM, and 280 mM at a scan rate equal to 20 mV/s.

Figure 5 illustrates the cyclic voltammetry tests carried out on the developed bioanode, varying the glucose concentration in a NaOAc buffer solution (pH 5.0). The enzymatic activity on the substrate, detectable from the higher currents registered during oxidation and reduction phases, started at high glucose concentration (i.e., 280 mM), whilst it was not visible at lower concentrations. This finding was also corroborated by performing chronoamperometry tests on this bioelectrode.

#### 3.4.3. Biocathode Cyclic Voltammetry

**Figure 6:**
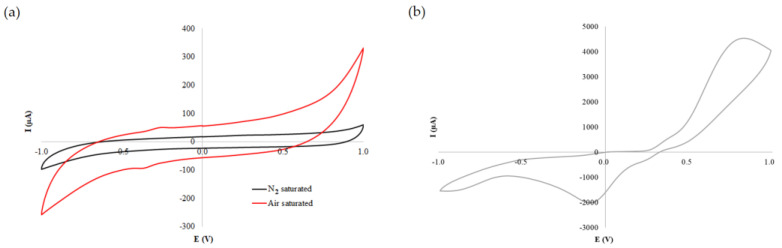
(**a**) Cyclic voltammetry registered for the biocathode in N_2_-saturated (black line) and air-saturated (red line) NaOAc buffer solution (pH 5.0) at a scan rate equal to 20 mV/s. (**b**) Cyclic voltammetry registered for the biocathode in air-saturated NaOAc buffer solution (pH 5.0) with catechol as a mediator at a scan rate equal to 20 mV/s.

Figure 6a depicts the registered CV for the biocathode in a nitrogen-saturated and air-saturated phosphate buffer solution. The catalytic process of oxygen reduction involving laccase is very slow, therefore clear catalytic curves are formed at very low rates of potential change, according to the literature [55]. Hence, the CV tests were conducted at a relatively low scan rate (20 mV/s). As visible from Figure 6a, the laccase voltammogram in an air-saturated solution (red line) is clearly wider than the nitrogen-saturated one (black line), demonstrating the catalytic activity of the biocathode when the fuel (oxygen) was dissolved in the solution.

Moreover, the kinetics and analytical features of the biocathode were tested in the presence of the redox mediator catechol, as shown in Figure 6b, similar to the behavior described in [56]. It must be highlighted that the current output of the biocathode significantly increases in the presence of the catechol mediator, with the electron transfer between the laccase enzyme and the support being more efficient. Furthermore, the ratio between the anodic current peak (*I_p,a_*) and the cathodic current peak (*I_p,c_*) is $\frac{I_{p,a}}{I_{p,c}}\;\sim \;2$, as also indicated in ref. [57].

#### 3.4.4. Chronoamperometry

**Figure 7:**
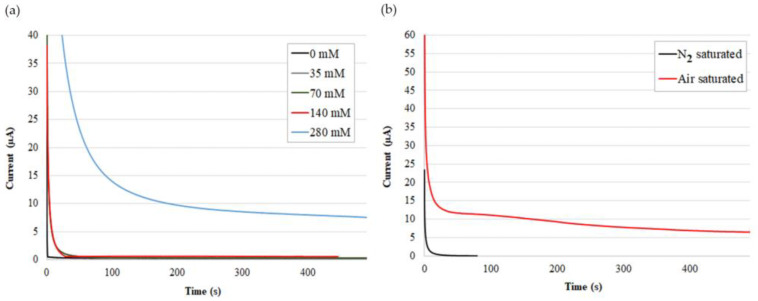
Chronoamperometry registered at 0.1 V for: (**a**) bioanode varying the glucose concentration from 0 mM to 280 mM in a NaOAc buffer solution (pH 5.0); (**b**) biocathode in N_2_-saturated and air-saturated NaOAc buffer solution (pH 5.0).

To quantify the amount of current passing through the electrodes, chronoamperometry (CA) tests were realized both for the bioanode and biocathode, as illustrated in Figure 7. Specifically, Figure 7a reports the CA at different concentrations of glucose. Only the bioanode working with 280 mM glucose concentration showed a noteworthy steady-state current (that reached about 6 µA). At lower concentrations, the steady-state current was lower than 1 µA (varying from 0.5 µA at 140 mM to 0.25–0.3 µA at lower glucose concentrations), demonstrating a very low electron transfer through the bioanode. Concerning the biocathode, Figure 7b highlights that the registered steady-state current in the presence of substrate (air-saturated conditions) reached 6 µA, whilst in nitrogen-saturated solution the current tended very quickly to zero. These considerations, together with the results of the CV, demonstrate that the direct electron transfer mechanism from the enzymes to the PV15 support works for both the implemented bioelectrodes. The results obtained in terms of current densities, as demonstrated by means of CV and CA electrochemical characterization, are in line with the literature for both the GOx bioanode [58,59,60,61,62] and the laccase biocathode [57,63].

#### 3.4.5. Open Circuit Voltage Test

To evaluate the performance of the bioelectrodes in terms of voltage stability over time, open-circuit voltage (OCV) was registered for six weeks.

For what concerns the GOx bioanode, a stable voltage output with a maximum OCV value of 120 mV vs. Ag/AgCl was registered during the six-week analysis campaign.

As for the laccase biocathode, the first measurement (performed at week 0) showed an OCV value of about 350 mV vs. Ag/AgCl. After 4 weeks, the laccase biocathode presented an overall potential drop of 130 mV (−37% reduction relative to the initial value registered at week 0) in a month, corresponding to a weekly reduction rate of just over −8%. In the following week (i.e., week 5), there was a further reduction of 50 mV vs. Ag/AgCl, equal to −22.7% compared to the value of 220 mV vs. Ag/AgCl registered at week 4, with a significant increase in the weekly reduction rate. As regards the last week (week 6), the laccase biocathode showed a maximum voltage of approximately 110 mV vs. Ag/AgCl, with a reduction of 60 mV compared to the previous test of week 5 (−35%). A global reduction of about −68% was exhibited compared to the OCV measured in the initial state (350 mV). Indeed, to increase the biocathode potential and to avoid the mediator overpotential, direct electron transfer (DET) concerning the laccase enzyme has been strongly investigated to date. The obtained result proves a significant enhancement in the biocathode stability, in terms of voltage output over time. This aspect, as discussed in the literature [25,64,65,66,67], represents one of the crucial issues limiting the development of biofuel cells, together with the needed increase in the produced power density. Therefore, this work can be considered a great starting point for future advances in the EBC field.

## 4. Conclusions

This study highlights the development and biochemical characterization of electrodes intended for the design of an EBC based for the first time on commercial support (Sigracell^®^ PV15 fluoropolymer), on which the GOx and laccase enzymes were covalently immobilized for the anode and cathode electrodes, respectively. Two different immobilization techniques specifically suitable for the two considered enzymes were employed, allowing good immobilization yields (>80%) and long-term stability of the immobilized enzymes for several operating cycles. The procedures were optimized in terms of enzyme concentrations required to reach both good values of recovered activity for the immobilized enzyme relative to its free form and the greatest enzymatic activity for units of area (U/cm^2^), thus finding the right quantities of the enzyme to be immobilized, avoiding waste of catalyst, and allowing economic savings. The electrodes loaded with the enzymes were then characterized by SEM and FT-IR/ATR spectroscopy, and their functionality was tested by electrochemical analysis. The latter demonstrated that a direct electron transfer mechanism is operative for both bioelectrodes, whose stability over time was also monitored for six weeks. Hence, the identification of efficient immobilization protocols, together with the use of a low-cost commercial conductive polymer for the preparation of functioning bioelectrodes, provides the basis for the progress in EBC technology and its possible production for biomedical devices and industrial applications for power generation.

## Figures and Tables

**Table 1:** Characterization of the different laccase-PV15 films: immobilization yield, specific activity, and enzymatic activity.

Film	Free Laccase (mg/mL)	Administered Laccase (mg)	Immobilized Laccase (mg/cm^2^)	Immobilization Yield	Specific Activity (mU/mg)	Recovered Activity	Enzymatic Activity (mU/cm^2^)
1	17.5	8.8	3.7 ± 0.1	42%	1.1 ± 0.1	0.11%	4.1 ± 0.5
2	8.75	4.4	2.1 ± 0.2	48%	2.3 ± 0.1	0.23%	4.9 ± 0.7
3	2.0	1.0	0.80 ± 0.04	80%	13.9 ± 0.2	1.4%	11.1 ± 0.7
4	1.0	0.5	0.47 ± 0.05	94%	35.3 ± 0.4	3.6%	17 ± 2
5	0.40	0.20	0.18 ± 0.01	90%	128 ± 4	12.9%	23 ± 2
6	0.10	0.05	0.050 ± 0.003	93%	167 ± 3	16.9%	7.7 ± 0.6
7	0.040	0.02	0.019 ± 0.003	94%	360 ± 50	36.8%	7 ± 1
8	0.020	0.01	0.010 ± 0.001	96%	450 ± 40	45.2%	4.8 ± 0.9

## Data Availability

The datasets used and/or analyzed during the current study are available from the corresponding author upon reasonable request.
